# New York Odontological Society

**Published:** 1892-09

**Authors:** 

**Affiliations:** New York Odontological Society


### Reports of Society Meetings.

### NEW YORK ODONTOLOGICAL SOCIETY.

A REGULAR meeting of the New York Odontological Society was held on Tuesday evening, May 17, 1892, at the New York Academy of Medicine, No. 17 West Forty-third Street, New York City; the President, Dr. Woodward, being in the chair.

The minutes of the previous meeting were read and duly approved. Professor Carl Heitzmann then read a short paper entitled, "Demonstration of the Reticulum in Dentine with Low Powers of the Microscope."

(For Dr. Heitzmann's paper, see page 629.)

*Professor Heitzmann.*—I must remark that this stain fades. This specimen was very much plainer than it is to-day. Only experts should look at this. I had not the slightest idea that the specimens would fade in glycerin until Dr. Howe called to see me, and then I noticed that they were much faded in appearance.

*The President.*—Any one wishing to examine the specimens may now do so, after which the question will be open for discussion.

### DISCUSSION.

*Dr. Howe.*—At Dr. Heitzmann's invitation I went to his laboratory, and saw in these specimens, very clearly, what he describes as the reticulum. But I do not feel competent to express an opinion as to what it is that I see. The specimens are perfectly clear to my eye, and would appear to confirm what Dr. Heitzmann says; but I am willing to be instructed.

*Dr. S. E. Davenport.*—I feel that this Society is under obligations to Drs. Heitzmann and Bödecker for presenting before us this evident step in histology. There is, to my mind, no more interesting study than that of histology, and dental histology particularly. It was my good fortune to have a short course in Dr. Heitzmann's laboratory some years ago, and although I was as untrained as any boy could be in the use of the microscope, I was perfectly converted to the idea that the reticulum not only existed, but that I could see it plainly in many specimens. But those specimens were shown under a very high power, and were consequently not as plain, by a great deal, as those which are presented to-night. I

now feel that if those gentlemen throughout the country who have all along objected so strenuously to the idea that there was a reticulum in the dentine could have the privilege of seeing these specimens they would say, "Certainly there is a reticulum," for without doubt the metallic particles from the amalgam filling are deposited in the structure of the dentine at some distance from the periphery of the cavity.

*Dr. Allan.*—As Professor Heitzmann said some fourteen years ago, this new discovery as to the reticulum was announced by Dr. Bödeker, and certain specimens shown to prove his position. In a paper read by Professor Heitzmann last year before this Society, he presented the subject of the reticulum in protoplasm, and his claims as to its discovery; that is a question on which I never had a controversy with him at all, but which I propose to leave him with his peers, who, by the way, give him very little credit for its discovery. If you will hunt through the books on histology and physics, you will be astonished at the few times Professor Heitzmann's name is mentioned, and the very little credence given to the fact that he discovered the reticulum at all, or had any connection with it. But that is not the question before us. The only dispute I have ever had with him is the one relating to the reticulum in dentine and enamel, which I have always denied, and now, after looking at his specimens, more positively deny than ever. More strenuously than ever I defy Dr. Heitzmann and Dr. Bödecker to accept the invitation extended to them by the Society in a carefully-drawn resolution, worded by our worthy president at that time,—Dr. Howe, who retained *my* explanation, protoplasmic reticulum,—inviting them not to submit the specimens to experts chosen by the Society alone, but to experts chosen by both the Society and Professor Heitzmann. They were invited to coöperate with the Society to prove their position by showing their specimens to those capable of judging what they could see.

If any of you gentlemen have a question in dentistry that is important, you do not go to a committee on microscopy; nor does an astronomer pick out a geologist if he has a question of the sidereal world to solve. Why can we not have the specimens submitted to those who, from their position and acquirements, are able to settle this matter forever? Any decision that the Society will agree to that will simply open the door to a careful examination of these slides will be satisfactory to me. Dr. Miller, of Berlin, has been most anxious to see these slides and these specimens, but he has never been able to do so. He called on Drs. Abbott, Heitzmann, and Bödecker, but he never could see any slides or specimens, as he was told that the lenses were either out of order or the gentlemen did not have them at home at that time. Drs. Andrews, Black, and Sudduth deny that this reticulum exists. Though they have tried diligently, they have never been able to see it. Now, whether I am right or wrong is very easily proven. This question has been before the dental public for fourteen years, and outside of New York you will find no one who accepts these views or teaches them. What is the meaning of it? They cannot get slides to show this reticulum. There is no proof of it, therefore they do not teach it. I deny that the reticulum is shown in those specimens.

*Dr. Bödecker.*—I think this is nothing but a personal matter between Professor Heitzmann and Dr. Allan. When Dr. Miller was visiting me, I gave him forty or forty-six specimens. My lenses have never been out of order when a microscopist called at my house. If any of the gentlemen will come to Dr. Heitzmann's laboratory, I am certain they are at liberty to examine anything they wish. Dr. Heitzmann will go to the laboratory any time and allow them to examine all the specimens.

*Dr. Hart.*—I think we have been wasting about fifteen or twenty minutes trying to discuss matters that took place before this Society a long time ago. How would Dr. Allan account for the stain exhibited by Dr. Heitzmann taking place within the tooth and a space being crossed by that stain, if it were not for the reticulum described by Dr. Heitzmann? If we discuss the paper, and do not refer to facts which took place long ago, more important conclusions can be arrived at.

*Dr. Allan.*—I wrote to Dr. Miller immediately after what Dr. Bödecker told me at a late gathering of dentists, and I have Dr. Miller's letter, in which he tells me that he has some six unimportant specimens of Dr. Bödecker's, none of them even claiming to show the reticulum, and that when he called on the latter his oil-immersion objective was missing. As to Dr. Hart's statement, I want to say that this is merely a matter of demonstration,—simply that and nothing more. I do not care for any theory not backed by proof. You can make a theory to meet anything. I deny that there is a reticulum present, and what I assert is that skilled microscopists will say so when they examine the proof preparations. The finer branches of the canaliculi would explain all that Professor Heitzmann alluded to. There is no difficulty in accounting for living matter throughout the entire basis-substance of the dentine. It is not necessary to suppose that there is a reticulum there. I only want to say one word more. I again challenge Professor Heitzmann and Dr. Bödecker to submit, in any way that this Society may choose, their specimens before such a committee as the Society and they jointly may determine, and let us then have the matter decided for us.

*Dr. Walker.*—Can I ask Professor Heitzmann and Dr. Allan one question? Can either of them tell me whom they consider dental microscopists in this country? Then I will accept the challenge as offered by Dr. Allan; I am prepared to do it.

*Dr. Allan.*—I suppose Dr. Black is a dental microscopist, also Dr. Andrews and Dr. Sudduth; but I assure you that it is not necessary for a man to be a dentist to be an expert microscopist and histologist. This is a question of demonstration in histology. On that broad basis I stand. In the Twenty-third Street College, the Johns Hopkins University, or wherever you go, there are trained microscopists enough who will answer this question for us.

*Dr. Walker.*—Can Professor Heitzmann name any one?

*Dr. Heitzmann.*—Dr. Walker, modesty forbids it.

Dr. E. H. Stebbins, of Shelburne Falls, Mass., then read the paper of the evening, entitled "Argenti Nitras as a Remedy in the Treatment of Pyorrhœa."

(For Dr. Stebbins's paper, see page 659.)

DISCUSSION.

*Dr. Brockway.*—Do you mean by treating a few teeth at a time, at one sitting?

*Dr. Stebbins.*—Yes, at one sitting.

*Dr. Allan.*—In the use of nitrate of silver for pyorrhœa, is one application of the powder sufficient?

*Dr. Stebbins.*—Yes, usually.

*Dr. Allan.*—Does it show as much discoloration on the necks of the teeth as in cavities?

*Dr. Stebbins.*—The compound that it forms with the organic matter in the cementum causes it to remain there. It is not so dark because you have not so much of the organic matter.

*Dr. Allan.*—Do you not have necrosis of the soft tissues?

*Dr. Stebbins.*—Yes; but it will take that all away. It will slough, and the gum, in every case I have seen, becomes hard. You press on it, and there is no pus exuding.

*Dr. Allan.*—There is no destruction of good tissue?

*Dr. Stebbins.*—The operation of the nitrate is superficial, so if it be applied to the gum it will not penetrate deeply,—not so far as to destroy more living tissue than necessary.

*Dr. Allan.*—Did you say it will remove tartar?

*Dr. Stebbins.*—No; I said that the tartar will not re-deposit. It will not remove the tartar that is there. In the case I mentioned the tartar would re-deposit, but now it does not do so to any amount.

[Dr. Stebbins produced a boy and girl on whose teeth he had used the nitrate of silver. In the boy, the temporary second molars, the upper and one of the lower teeth, were treated six years ago last March, and have not been treated since.]

I am talking of the deciduous teeth now. The approximal cavities were treated four years ago; the patient would pick them, and the cavities increased in size and have been again treated. I have put in gutta-percha in some to keep them from being interfered with by the patient. You may examine this boy if you wish.

*Dr. Bödecker.*—About eighteen years ago I experimented a great deal with nitrate of silver in different ways; not in the way of treating caries, but in pyorrhœa alveolaris, abscesses, necrosis, tumors, etc. The method of application varied from that described by the author of the paper. At that time I came upon a method by which you can carry the minutest quantity of a crystal of nitrate of silver into any spot you desire. If you take a very fine wire of platinum and make a little loop on the end, warm that loop in the alcohol lamp, and then dip it into powdered nitrate of silver, and melt whatever sticks to this loop, you can carry the nitrate of silver into any place you please. It will dissolve anywhere where there is moisture. Furthermore, in treating cases with nitrate of silver, I pursued a little different course. In using it under the gum to overcome the difficulty experienced when the nitrate of silver comes in contact with the tongue or gum, you do not need to use the paper, because paper will not prevent it on account of the moisture; the nitrate of silver will creep under the paper. Take a glass of salt water, and let the patient rinse the mouth before using the nitrate of silver; then dry thoroughly with paper the place which you want to cauterize, and apply the nitrate of silver. If anything flows out the salt water will immediately coagulate the nitrate of silver, and form an insoluble chloride of silver. Let the patient rinse the mouth with the solution of salt water after the application of the nitrate and no harm will be done to the surrounding tissues.

*Dr. Stebbins.*—In this little girl's teeth the second inferior temporary molars were treated six years ago. They have large cavities now, and when I get through this evening I shall put some kind of soft filling in those cavities, and then she will have no more trouble about the progress of decay.

*Dr. Bödecker.*—I have been asked to explain my experience with the nitrate of silver in pyorrhœa alveolaris. I would state that my results with it have not been quite as satisfactory as with some other methods I have employed. My present method of treating most of the cases of pyorrhœa alveolaris is that I use the spray apparatus with some antiseptic. With this I have had more success than with anything else; at the same time treating those cases with a solution of iodide of zinc, the prescription of which was given to me some fifteen years ago by Dr. Northrop, and I must thank him again for it now. With Dr. Northrop's permission I will give the prescription to the society. It is a saturated solution of sulphate of zinc, a saturated solution of iodide of potassium, equal parts, which in turn is saturated with the crystals of iodine.

*Dr. Hodson.*—I would like to ask Dr. Stebbins what method he takes to obviate the possible effects of this material upon a fully or nearly exposed pulp in the first teeth. What happens if it is exposed?

*Dr. Stebbins.*—That brings to my mind a case that came into the office Saturday. Last July a lady brought her little boy, about three years old, to me. He had been suffering with toothache that kept him awake, and she had heard that I was treating children's teeth, and so brought him in to see if anything could be done. The upper incisors and cuspids were decayed. Some had large cavities, and some not very large. The child had only sixteen teeth erupted. The second temporary molars were not through. The upper molars were decayed badly, and probably exposed pulps caused the pain. I did not ascertain whether the pulps were exposed or not. I treated all those teeth with the nitrate, and he has not had the toothache since. That was about ten months ago. One of the molars has a pulp-tumor. The pulp of the other is dead. When I get home I shall remove that tumor, probably by cauterizing it, and then prepare and fill the pulp-chambers. I have been asked about the application of the salts. You can see by this instrument. It is rather crude, but it will hold the wooden points, and it will reach the posterior side, or any part of the teeth. If I want to go to the side of the tooth in pyorrhœa, I make it very slender, and I have the salts pulverized. I keep them in an open dish, so I can dip the point in. I would just moisten it, touch it on the salts, apply it to the tooth, be sure that it is thoroughly covered, hold it there a minute, then syringe it out. You do not have to keep the mouth perfectly dry. I have never known a case where the overflow of the dissolved salts caused any trouble. If it were a deep cavity, and I wanted to hold it a little longer, I would place the salts in, cover with a piece of cotton, and let it stay there five minutes; then draw it out and dismiss the patient. In pyorrhœa you will learn how much it takes by trying better than I can tell you. Carry it down as far as the tooth is exposed. In a few days I find the gum hard, where it was formerly soft and fluctuating. In the case of a patient I know, the left central and lateral incisors and cuspid were affected. That patient had lost many teeth with pyorrhœa, and these were very loose and sensitive. I put the salts around them. He came home from the Legislature a week later, and I pressed the gums, but found them perfectly hard.

*Dr. Brockway.*—Some dentists speak of using a solution of the salts. Is there any advantage in that?

*Dr. Stebbins.*—I think not; the salts, with the organic matter, form an insoluble compound.

*Dr. Hodson.*—Then Dr. Stebbins does not care whether the pulps are exposed or not when he makes this application?

*Dr. Stebbins.*—Dr. Howe said it was a good way to destroy the pulp. It will destroy the pulp in adults. In two cases I destroyed it by a too close application of the agent. I have not tried much to keep exposed pulps of deciduous teeth.

*Dr. Hodson.*—I knew it would destroy the pulp, but the question arose whether you would use it to relieve pain in a child's tooth.

*Dr. Stebbins.*—Yes; the cases that came to me last Saturday were those where the children had pain.

*Dr. Brockway.*—I simply wish to say that I was very much impressed with Dr. Stebbins's article in the INTERNATIONAL DENTAL JOURNAL last October, and I was the more impressed because I have been in the habit of using nitrate of silver myself occasionally for many years. I am quite ashamed to mention this fact, inasmuch as I did not have courage enough, or did not use it understandingly enough, to go on and secure decided results; but I have for the past twenty-five years occasionally touched softened spots, especially on the buccal surfaces of the molar teeth, with nitrate of silver with the hope of retarding decay. I have done it almost empirically, and with good results; but, as I say, I have not had courage enough to carry the treatment out as I should have done, and I was, therefore, especially pleased with Dr. Stebbins's article, because it confirms what little experience I have had and strengthens it.

Since reading the article I have used the nitrate of silver in many cases, and although it is too soon to speak with much positiveness of the results, I have the greatest faith in the treatment. If it will do anything like what is claimed for it, you will agree that it is one of the most valuable contributions to dental knowledge that we have had from any source whatever. I shall proceed to use it in cases of pyorrhœa, because I have great faith in the statements and illustrations which Dr. Stebbins has given.

*Dr. Howe.*—I suppose there is no doubt but that the use of nitrate of silver is old, and I think there are many who could say, as Dr. Brockway has, that they have used it. But it has remained for Dr. Stebbins to bring out the scientific application of it and demonstrate what it was capable of doing. When I saw Dr. Stebbins last summer in Boston, and saw some of the results that he presented there, I was very much impressed with the importance of his contribution. When I came home, I wrote two or three letters to mothers of children who had brought their babies to my office some months previously, when I had been unable to do anything for them whatever, because of the sensitiveness of extensively decayed teeth and the nervous condition of the little patients. I asked these ladies to bring their children back again, and let me see if I could not do something on a new line; and I did apply silver nitrate to these sensitive decaying molars in October. I have recently seen those children's teeth, and they are as black as they can be in the decayed places, and as comfortable as if they never had been decayed. That is only a record of six months, but it is a confirmation of the plan of treatment, as far as it goes. I have used it for the treatment of bad cases of pyorrhœa, but I can say nothing as to results, because the cases are too recent; but I have the greatest confidence that this contribution of Dr. Stebbins's, especially in giving us another means of arresting decay, is one of the most valuable that we have had in many years.

A vote of thanks was tendered to Dr. Stebbins for his extremely interesting and instructive essay.

*Dr. Stebbins.*—I thank you very much for your kindly reception of me and my paper, and the kindness shown me. I should feel very much out of place to come here to New York if I were not assured that my field is a large one and of deep interest to every practitioner, and by means of which we may relieve cases in which we were unable to do anything before, and that without pain or expense to the patient. I trust it will do good, and that is what I am here for.

*Dr. Howe.*—I wish to offer the following resolution for action by the Society:

"*Resolved*, That in view of the variable quality and indefinite properties and composition of our compound filling-materials, this Society desires to record its interest in the improvement of compounds for filling teeth, and would invite the co-operation of pharmacists, chemists, and metallurgists, as well as dental manufacturers, in all reasonable efforts to improve and make uniform such cements and alloys as are available for our purposes. We believe that investigation and enterprise in these directions would be abundantly remunerated, and that progressive improvement and professional sentiment would be stimulated by the publication of the formulas of all compound preparations for our use. We recommend that this subject be referred to a committee of this Society."

Resolution seconded and adopted.

Adjourned.

John I. Hart, D.D.S.,
*Editor New York Odontological Society.*

